# Endovascular Diagnosis and Successful Treatment of Massive Gastrointestinal Hemorrhage in Children

**DOI:** 10.4274/balkanmedj.2018.0322

**Published:** 2018-09-21

**Authors:** Abdulkerim Temiz, Murat Gedikoğlu, Semire Serin Ezer, Pelin Oğuzkurt, Akgün Hiçsönmez

**Affiliations:** 1 Department of Pediatric Surgery, Başkent University School of Medicine, Ankara, Turkey; 2 Department of Radiology, Başkent University School of Medicine, Ankara, Turkey

Severe gastrointestinal hemorrhage is usually related to Meckel’s diverticulum, intussusception, or peptic disease in children (1). Visceral vascular anomalies very rarely cause upper gastrointestinal bleeding (2,3). We present four pediatric cases with massive upper gastrointestinal bleeding that were diagnosed and treated with endovascular techniques.

Four patients aged from 6 to 15 years presented with a massive upper gastrointestinal bleeding. Written informed consent was obtained from all patients. All patients underwent endoscopy. Duodenal ulcer and hemobilia were detected in two and one patient, respectively. The bleeding source could not be detected by endoscopy in one patient. Although duodenal ulcer bleeding was stopped using adrenalin injection, massive upper gastrointestinal bleeding recurred after 1 week in one patient. Superselective celiac angiography was performed, and the bleeding sources were determined in all patients. Angiography was performed under the conditions of emergency intervention.

All procedures were performed under general anesthesia. Arterial access was obtained by sonographic assistance in every patient via puncture of the common femoral artery. A micropuncture set and 4F systems were used. Embolization of bleeding was performed uneventfully using pushable and/or detachable coils. Gastroduodenal artery pseudoaneurysms were detected in two patients with duodenal ulcer (Figure 1). The bleeding was found from a branch of the right hepatic artery in the patient with hemobilia (Figure 2). Bleeding from the left gastric artery was detected by angiography during the active bleeding period in the patient in whom the bleeding source could not be detected by endoscopy. Coil embolization was performed successfully to stop the bleeding in all patients. Clinical and laboratory findings were stable after embolization. There was no recurrence of bleeding during the follow-up periods.

Although several diagnostic techniques have been described for the determination of the cause and source of bleeding, endoscopy remains a reliable and an effective diagnostic tool to establish the cause of upper gastrointestinal bleeding. However, endoscopy is sometimes inadequate, especially in cases of intermittent or obscure bleeding (4).

Endoscopy is generally useful for therapeutic interventions; however, it may be insufficient in some cases. Surgical treatment, which carries a high risk of morbidity, may be necessary. In such cases, selective visceral angiography facilitates the diagnosis and may also facilitate the use of endovascular treatment. As such, angiography may reduce surgical morbidity rates. The success rates of endovascular treatment in a large adult series were reported to be as high as 80% (5).

Accessing the pediatric vessels can be more challenging because of their smaller size and the vulnerable nature in contrast to those of adults. Therefore, arterial access was obtained under sonographic guidance in all cases in the present study. The small vessel size also constrains the choice of catheters.

In conclusion, endoscopy and endoscopic treatment are still the gold standard of diagnosis and therapy for upper gastrointestinal bleeding in children. Selective visceral angiography reduces morbidity and mortality when it is performed by experienced hands, and therefore, it must be considered as an alternative when endoscopy is insufficient for diagnosis and treatment.

## Figures and Tables

**Figure 1:**
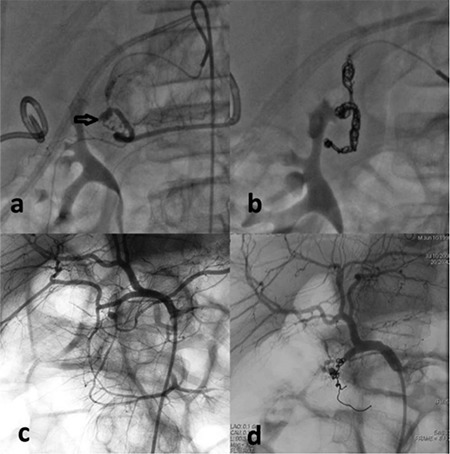
a-d. Gastroduodenal pseudoaneurysm was detected by celiac angiography. Massive bleeding was observed in a 13-year-old boy (a) and a 10-year-old boy (b). Embolization stopped the bleeding, respectively (c, d).

**Figure 2:**
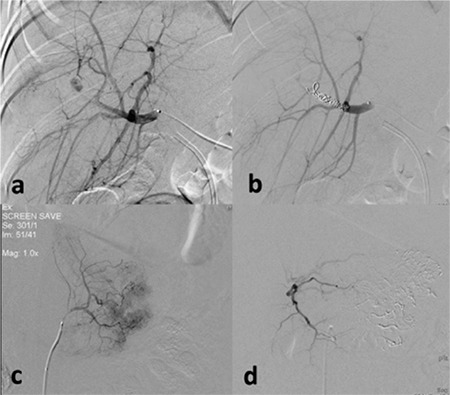
a-d. Superselective hepatic angiography demonstrated hepatic artery aneurysm and bleeding in a 6-year-old boy with hemobilia (a). Coil embolization was performed (b). Active bleeding from the left gastric artery was detected by angiography in a 15-year-old boy (c). Embolization stopped the bleeding (d).
